# Image-Based Assessment of External Quality Traits in Chinese Bayberry (*Myrica rubra*) Using a Deep Learning Detection and Multi-Trait Scoring Framework

**DOI:** 10.3390/foods15162852

**Published:** 2026-08-15

**Authors:** Mengting Wang, Lijia Lei, Hongdou Liang, Jiawei Jiang, Yalu Chen, Yunyan Hua, Tao Ma, Wona Ding, Xu Li

**Affiliations:** Ningbo Key Laboratory of Agricultural Germplasm Resources Mining and Environmental Regulation, College of Science and Technology, Ningbo University, Cixi 315300, China; wangmengting@nbu.edu.cn (M.W.); zhengqingbo@zwu.edu.cn (L.L.); 201816213@mail.sdu.edu.cn (H.L.); lixu@ouc.edu.cn (J.J.); 15258538527@163.com (Y.C.); huayunyan2000@163.com (Y.H.); matao08@163.com (T.M.); dwn@zju.edu.cn (W.D.)

**Keywords:** Chinese bayberry, deep learning, postharvest quality, multi-trait scoring

## Abstract

Chinese bayberry (*Myrica rubra*) is an extensively popular fruit with unique flavors and high nutritional values. A precise and efficient method for assessing external quality traits of Chinese bayberry is critical for machine automatic sorting to ensure the quality and increase economic value. Here, we developed an automatic evaluation flow based on deep learning detection and a multi-trait scoring framework for postharvest quality grading of Chinese bayberry. Firstly, the YOLOv11 framework was applied to detect the Chinese bayberry fruits, and the network consists of a CSPDarknet-based backbone with C3k2 modules, an SPPF block, a bidirectional PANet neck, and three decoupled detection heads. The experimental results showed a precision of 0.9991 and a recall of 1.0000 for the waxberry class (*mAP*@0.5 = 0.9950, *mAP*@0.5:0.95 = 0.8632). Furthermore, the quality scoring model was built on a modified AlexNet backbone that provided more stable training and better accuracy compared to the other seven CNN models. The R^2^, MAE, and RMSE showed the close alignment between predicted and reference values, revealing reliable estimations of our six fruit-quality traits, including radius, color depth, color class, color uniformity, surface injury area ratio, and plumpness. This self-developed flow provides a comprehensive and effective assessment method to improve fruit quality and economic value, which further facilitates automated agricultural production.

## 1. Introduction

Chinese bayberry (*Myrica rubra*), as one of the most distinctive and popular berries, is primarily cultivated in the subtropical regions of China, with an annual fresh fruit output value reaching 6.3 billion yuan [1,2]. The mature fruit displayed an attractive red color, and its edible portion consists of papillary protrusions that are soft and full of juice, giving it a unique flavor [3]. Moreover, Chinese bayberry possesses high nutritional value, being rich in natural bioactive compounds such as anthocyanins, phenolic substances, and organic acids [4,5,6,7]. Over 60% of Chinese bayberry harvest is sold as fresh fruit, predominantly targeting the premium market segment, where quality is a critical determinant of sales success [8]. Due to the lack of a leathery outer layer, Chinese bayberries are prone to suffering mechanical damage and decaying by fungi, which significantly reduces their storage life and commodity values [9]. Moreover, external physical characteristics (e.g., sizes, color) are also significant to the commodity value of Chinese bayberry [10]. To ensure the quality of fresh Chinese bayberries, manual quality assessment and grading are commonly employed post-harvest. However, manual quality detection highly depends on visual inspection and the tactile experience of workers, leading to strong subjectivity, low efficiency, and high losses [11]. The traditional manual sorting method is difficult to satisfy the demands of the expansion of the scale of Chinese bayberry cultivation, the increasing fruit market requirements, and the high-quality demand. Therefore, more efficient methods are needed to be applied in Chinese bayberry quality detection for reducing losses and assuring quality.

Computer vision provides a method for image-based feature detection to address rater bias in agriculture postharvest assessment [12]. It has been widely used in the fruit sector, exemplified by image-based fruit quality detection [13,14]. As a significant branch of artificial intelligence, deep learning (DL) has rapidly enhanced the efficiency of image-based analytics and has been applied to massive image information processing [15,16]. Many deep learning models have been broadly adopted across a wide range of fruit crops for type identification, coarse classification, and quality assessment, such as CNN (convolutional neural networks), YOLO (You Only Look Once), and RNN (Recurrent Neural Network) [17]. For instance, YOLOv11, MobileNetV3, and Mask R-CNN models were applied to process multi-angle and multi-pose datasets for fine-grained phenotyping of tomato, which had the potential for plant phenotyping estimation [18]. In addition, YOLOv11 was applied in coffee fruit maturity detection, peach inflorescence density estimation, and lychee pest detection, among other applications [19,20,21]. Moreover, Hadipour-Rokni et al. established a system for the detection of citrus fruit pests using CNN models (ResNet-50, VGG-16, AlexNet, etc.) and machine vision [22]. Meanwhile, the research paradigm has shifted from single-target recognition to collaborative multi-attribute analysis. An advanced model can always extract multiple quality parameters of fruits, such as color, size, shape, surface defects, and even internal characteristics (e.g., pH, soluble solids content, firmness) from sampled images in parallel [15,23,24]. To establish uniform standards and enhance efficiency, many models have been developed for fruit quality detection, such as apples, pears, and oranges [25,26,27]. For example, an automated apple-sorting machine embedded with a YOLO-based model was developed to sort the blemished apples from the high-quality fruit [28]. Compared with other fruits like apples, Chinese bayberry fruits are small in shape, and the features on their surface are more difficult to capture in intelligent quality assessment [29], yet Chinese bayberries are also a type of fruit that requires rapid and accurate quality assessment because of their increasing production and short storage life. However, it still lacks intelligent quality detection models with the capacity for small-target-feature capture and multi-dimensional quality evaluation in Chinese bayberry.

In this study, to address rater bias and enhance efficiency, we developed an automatic evaluation flow with a multi-dimensional quality evaluation framework for postharvest quality grading of Chinese bayberry. The flow excelled at capturing and identifying small-target agricultural features and provided a novel scoring pipeline covering six dimensions (radius, color depth, color class, color uniformity, surface injury area ratio, and plumpness), which could be applied to replace a portion of fruit quality trait prediction with manpower, especially for small berries, and offered potential solutions for the intelligent industry (e.g., identification of pests and diseases).

Compared with previous multi-task or multi-attribute fruit quality assessment frameworks, which typically evaluate one or two dominant traits (e.g., size or color) or rely on separately trained single-task models, the present framework differs in three respects: (1) it jointly predicts six heterogeneous external quality traits from a single shared backbone using an uncertainty-weighted multi-task loss that adaptively balances regression and classification objectives without manual tuning; (2) it is specifically tailored to small, densely clustered, low-contrast fruit targets, a setting in which detection is markedly more challenging than for larger fruits, such as apples or citrus; and (3) it integrates bounding-box detection with downstream trait scoring into a single deployable pipeline validated against eight alternative CNN backbones, providing practical guidance on backbone choice for small-scale agricultural datasets.

## 2. Materials and Methods

### 2.1. Data Collection

Chinese bayberry fruits were collected during different growth stages (40 d, 50 d, 60 d, and 70 d). The collection sites were from five orchards in Ningbo City, Zhejiang Province (121.33, 30.15; 121.40, 30.18; 121.24, 30.13; 121.46, 30.12; 121.28, 30.03). These collected fruits covered various morphological characteristics of Chinese bayberry during the process of fruit ripening, which provided a plentiful and comprehensive dataset for training. Images of these Chinese bayberry fruits were collected using a fixed color camera (Canon 200D Mark II DSLR, Canon Inc., Tokyo, Japan) under constant camera distance and focal length, and uniform lighting conditions in the laboratory. The resolution of Chinese bayberry images was 640 pixels × 640 pixels. A total of 1350 images were obtained for model training.

### 2.2. Data Annotation

Six quality dimensions were proposed according to the manual scoring procedure by experts, including radius, color depth, color class, color uniformity, surface injury area ratio, and plumpness. The radius of each Chinese bayberry fruit was measured manually, corresponding to each image. The radius ranged from 12 mm to 14 mm, which was identified as medium size (large size > 14 mm, small size < 12 mm). Fruit plumpness was calculated using the aspect ratio of corresponding images, where a value closest to 1 indicates a plumper fruit. Fruit plumpness was scored by multiplying the aspect ratio by 100. For instance, an aspect ratio of 0.98 yielded a plumpness score of 98. Then, three experts received standard training and independently scored the other four indicators (color class, color depth, color uniformity, surface injury area ratio) of each fruit image. The final reference score for each trait was calculated as the average of the individual scores. The scoring criteria were as follows: (1) Chinese bayberry fruits from different growth stages were classified into six colors (dark green, green, yellow-green, light red, deep red, and deep purple). Experts classified the colors of Chinese bayberry fruits based on the above standards; (2) For color depth, dark green scored 0 points, green scored 0 points, yellow-green scored 10–20 points, light red scored 30–60 points, deep red scored 60–80 points, deep purple scored 80–100 points; (3) For color uniformity, dark green, green, and yellow-green scored 0 points. The scores of red fruits were determined based on the proportion of the red area, for example, if the red area proportion of a Chinese bayberry fruit was 20%, then the score of color uniformity was 20 points; (4) For superficial decay/injury, the scores were calculated by the area proportion of a fruit without decay or injury, e.g., the decay or injury area accounted for 20% of the whole area of a fruit, then the score of superficial decay/injury was 80 points. Inter-rater agreement was evaluated using Fleiss’ kappa, with an average value of 0.83. Ten percent of the images were re-annotated after two weeks, and the test–retest reliability was 0.81.

### 2.3. YOLOv11-Based Detection

**Figure 1:**
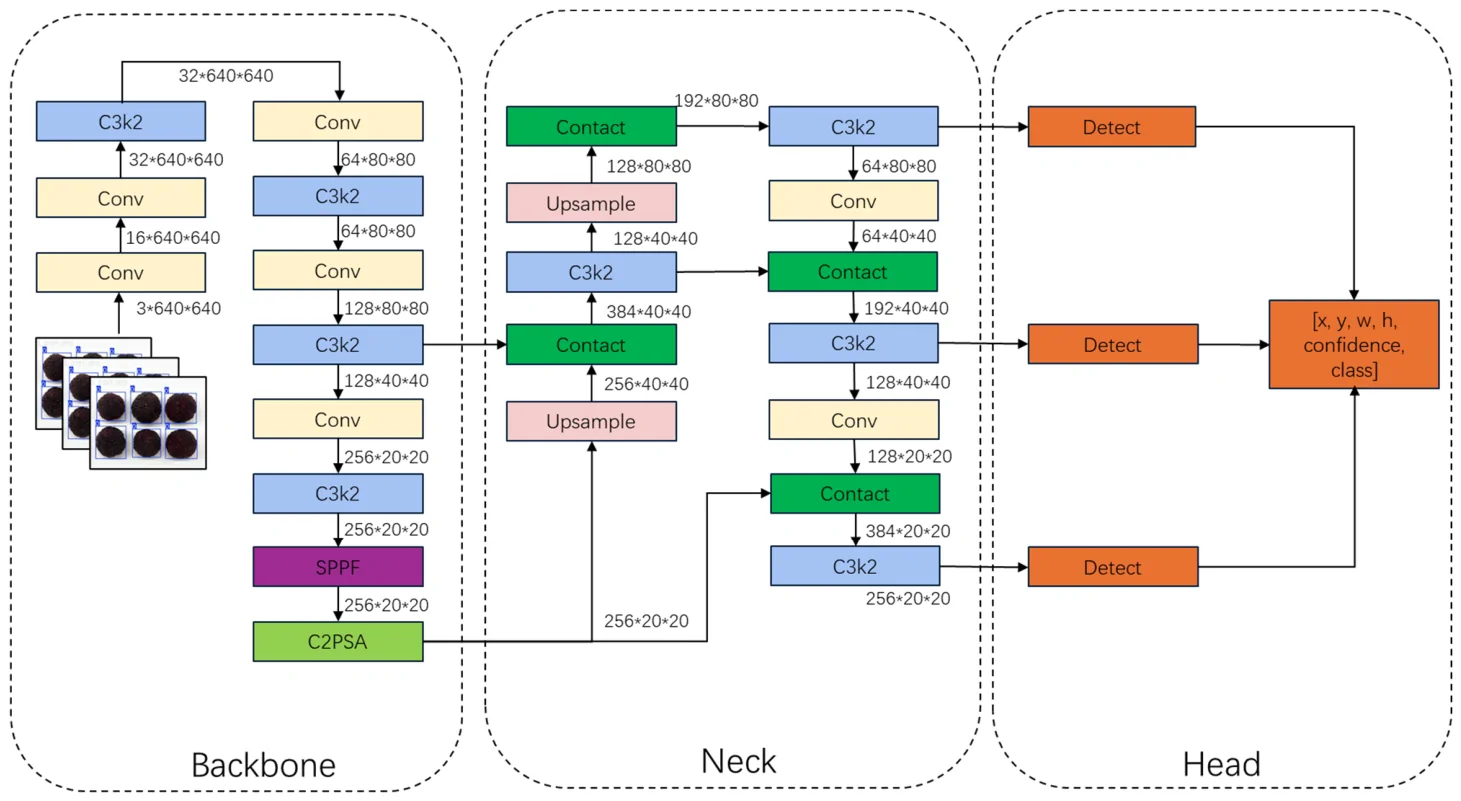
Architecture of the YOLOv11 model used for Chinese bayberry detection. The network consists of a CSPDarknet-based backbone, a bidirectional PANet neck, and three decoupled detection heads. The model outputs bounding boxes, confidence scores, and class labels for subsequent quality trait analysis.

The detection of Chinese bayberry fruits was performed using the YOLOv11 framework. The overall network structure is shown in Figure 1, consisting of a backbone for feature extraction, a neck for multi-scale feature fusion, and three detection heads for prediction at different spatial scales. The backbone builds upon the CSPDarknet design and incorporates C3k2 modules that replace standard convolutional blocks. Each C3k2 block contains a dual-branch structure with bottleneck residual units in one branch and a shortcut pathway in the other, which improves gradient propagation and feature reuse across stages. The backbone operates through five downsampling stages, reducing feature map resolution from 640 × 640 to 20 × 20 pixels while progressively increasing channel depth from 3 to 512. Strided 3 × 3 convolutions with padding are used to preserve spatial information and enlarge the effective receptive field.

To enhance the representation of fine surface patterns characteristic of Chinese bayberry, an SPPF (Spatial Pyramid Pooling Fast) module is integrated at the final stage of the backbone. This module aggregates multi-scale context using pooling kernels of 5 × 5, 9 × 9, and 13 × 13, enabling the network to better capture variability in fruit granularity, calyx scars, and superficial defects without increasing computational cost. The neck adopts an enhanced bidirectional Path Aggregation Network (PANet), which performs nearest-neighbor upsampling and concatenation-based feature fusion. This facilitates the transfer of high-level semantic information to higher-resolution feature maps, improving detection performance when fruits are partially occluded or occur in dense clusters. The final head employs a decoupled prediction structure with three output branches (P3, P4, and P5) responsible for detecting small, medium, and large fruit instances, respectively. Each branch predicts bounding box coordinates, objectness confidence, and class probability.

Fruit instances were extracted directly from the predicted bounding boxes rather than pixel-level segmentation masks; each detected bounding box was cropped from the original image to isolate an individual fruit region, and these crops were used to support downstream trait extraction for color, roundness, and injury assessment (Section 2.4). The network employs SiLU activation functions throughout to provide smooth gradient behavior and stable training dynamics. When deployed on modern GPU hardware, the model achieves real-time inference speeds while maintaining competitive accuracy for small-object agricultural detection tasks.

All architectural components described above, including the CSPDarknet backbone, C3k2 modules, SPPF block, bidirectional PANet neck, and decoupled detection heads, correspond to the standard Ultralytics YOLOv11 detection implementation (task: detect); no modifications were made to the detection network architecture itself, and no instance-segmentation (mask) head was trained or evaluated in this study. The methodological contribution of this study lies in the training recipe (data augmentation strategy and hyperparameters, Section 2.3.1) and its application to a small-target, densely clustered fruit detection task, rather than in altering the YOLOv11 architecture.

#### 2.3.1. Training Methodology and Implementation

The YOLOv11 model was trained using the Ultralytics implementation with a PyTorch v.2.13 backend. A total of 1350 images of Chinese bayberry fruits were annotated with bounding boxes (Ultralytics task: detect); no pixel-level segmentation masks were annotated in this study. The dataset was divided into a training set of 1080 images and a validation set of 270 images (8:2 ratio) [30]. All model development and training were conducted on a single GPU. This split was performed at the source-image level to prevent leakage into the downstream multi-task scoring stage described in Section 2.4.2.

To improve generalization under a limited sample size, several data augmentation strategies were applied. Mosaic augmentation (probability = 1.0) was used to merge four images into a single composite, increasing variation in fruit positioning and background conditions. Additional augmentation operations included random horizontal and vertical flipping (probability = 0.5), affine transformation, and color perturbation in HSV space (hue ±0.015, saturation ±0.7, and value ±0.4). MixUp (probability = 0.1) and copy–paste augmentation (probability = 0.1) were also used to enhance the robustness of detection under occlusion and fruit clustering.

Model optimization was performed using the AdamW optimizer, which decouples weight decay from gradient updates, with an initial learning rate of 0.01 and momentum parameters β_1_ = 0.9 and β_2_ = 0.999 [31]. For object localization, the loss function incorporates Complete Intersection over Union (CIoU) loss, which improves bounding box regression by jointly considering overlap area, center distance, and aspect ratio consistency [26]. Bounding box refinement was further enhanced using distribution focal loss (DFL), enabling more precise localization through discretized distance regression [32]. In addition, a standard classification loss was applied to distinguish Chinese bayberry instances from background regions. Training was conducted for 100 epochs with a batch size of 4. Model convergence was assessed by tracking box loss, classification loss, and DFL across training epochs, which together provide a comprehensive view of detection accuracy and optimization stability.

#### 2.3.2. Evaluation Metrics

Model performance was evaluated using four commonly adopted indicators for object detection tasks: precision, recall, *F*1-score, and mean Average Precision (*mAP*). Precision measures the proportion of correctly detected fruits among all predicted fruits, reflecting the ability of the detector to avoid false positives, whereas recall quantifies the proportion of annotated fruits that were successfully detected, reflecting sensitivity to true fruit instances. Their formulations are [33]:

(1) $$\mathrm{Precision}=\frac{TP}{TP+FP}$$

(2) $$\mathrm{Recall}=\frac{TP}{TP+FN}$$

where *TP*, *FP*, and *FN* denote true positives, false positives, and false negatives, respectively. Because precision and recall can trade off against each other, we also report the *F*1-score, which is the harmonic mean of precision and recall:

(3) $$F1=2\times \frac{\mathrm{Precision}\times \mathrm{Recall}}{\mathrm{Precision}+\mathrm{Recall}}$$

To provide a more comprehensive assessment of localization accuracy, we further calculated the mean Average Precision. For a fixed Intersection-over-Union (IoU) threshold τ, the IoU between predicted and ground-truth bounding boxes is where *B*_pred_ and *B*_gt_ are the predicted and ground-truth boxes [34]. For each class *c*, the Average Precision at IoU threshold τ is defined as the area under the precision–recall curve:

(4) $$\mathrm{IoU}=\frac{|B_{\mathrm{pred}}\cap B_{\mathrm{gt}}|}{|B_{\mathrm{pred}}\cup B_{\mathrm{gt}}|}$$

(5) $$AP_{c,\tau }=\int_{0}^{1}P_{c,\tau }(R)\;dR$$

and the mean Average Precision is obtained by averaging *AP* over all *C* classes:

(6) $$mAP_{\tau }=\frac{1}{C}\sum_{c=1}^{C}AP_{c,\tau }$$

In this study, the detection problem involves a single fruit class (*C* = 1), so *mAP* reduces to the *AP* of that class. We report *mAP*@0.5, computed at a fixed IoU threshold of 0.5, and *mAP*@0.5:0.95, computed by averaging *AP* values over IoU thresholds from 0.5 to 0.95 in steps of 0.05. Together, these four metrics provide a robust characterization of both detection reliability and spatial localization accuracy.

### 2.4. Multi-Task Quality Scoring Model (AlexNet)

**Figure 2:**
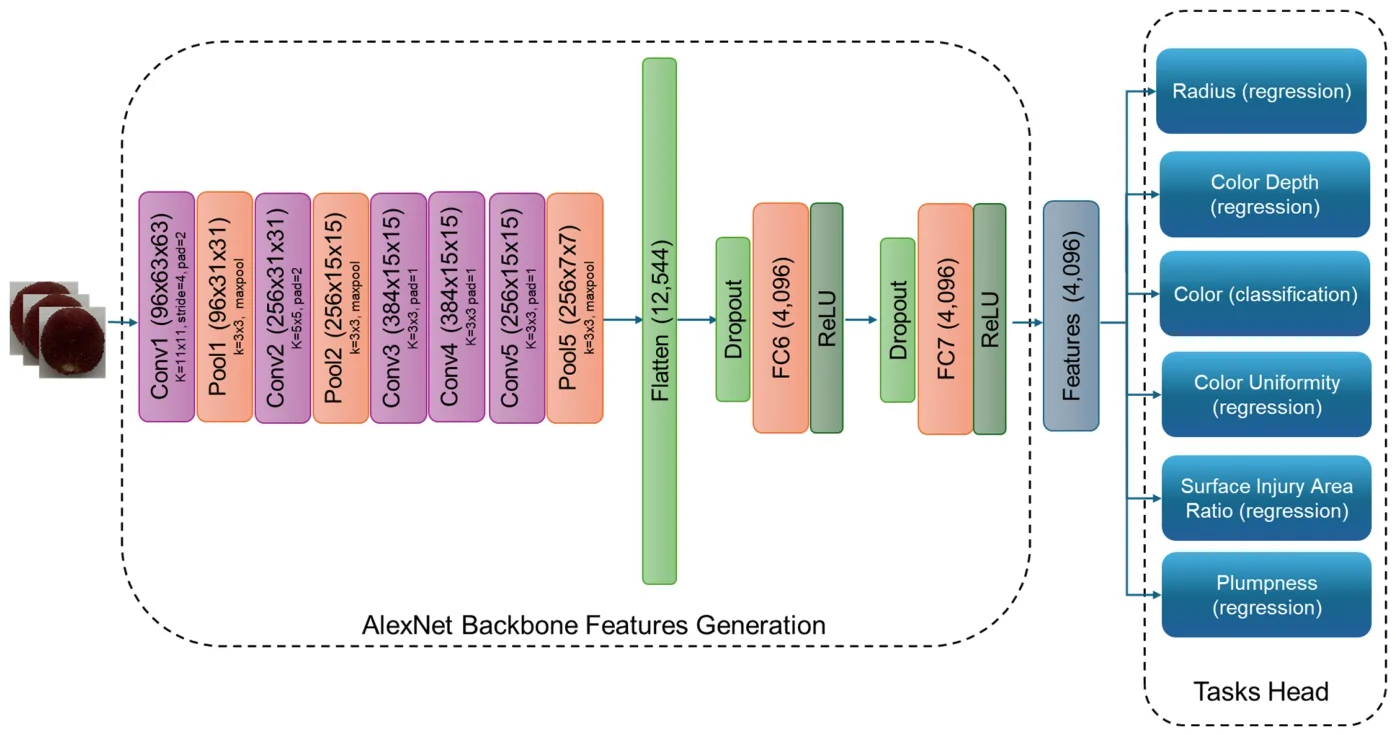
Architecture of the AlexNet-based multi-task fruit quality scoring model. A shared AlexNet backbone extracts a high-dimensional feature vector from each waxberry image. Six task-specific heads then predict radius, color depth, color class, color uniformity, surface injury area ratio, and plumpness.

The quality scoring model is built on a modified AlexNet backbone [35]. This structure was selected after comparing several common CNNs, where AlexNet provided more stable training and better accuracy for our six fruit-quality traits [36,37]. The network uses each cropped waxberry image as input and produces six predictions at once: radius, color depth, color class, color uniformity, surface injury area ratio, and plumpness. These traits describe both the appearance and the physical condition of the fruit, so the backbone must learn texture, color patterns, and shape cues in a balanced way [38,39]. As illustrated in Figure 2, the model begins with the standard AlexNet convolutional layers. These early layers capture coarse visual properties, such as berry outline and large color patches [40]. Deeper layers learn finer patterns, including pigment density, small surface defects, and the granularity that is typical of waxberry skins [41]. Compared with residual networks, AlexNet follows a straightforward feed-forward design without shortcut connections, which in practice gave more consistent results for our dataset size [37,42].

After the convolution blocks, the feature maps are flattened into a single vector. This produces a high-dimensional embedding, which keeps detailed texture information. Instead of using AlexNet’s original classifier, we replace it with a multi-task output module. This allows different quality traits to share the same backbone features while still having their own prediction branches. The multi-task module contains six task-specific heads. Five of these heads are configured for regression, used for predicting continuous values such as radius, color depth, uniformity, plumpness, and the injury ratio. The last head performs classification for the discrete color category. Each head includes a small number of fully connected layers, which adjust the shared features to the needs of each task. For example, the plumpness head focuses more on shape-related cues, while the color head relies more heavily on the chromatic information present in the embedding.

Using a single shared backbone has several advantages. First, it avoids training multiple models, which reduces computation and makes deployment simpler. Second, the different heads can benefit from each other because some visual properties are correlated. Traits like color depth and injury level often co-occur, and learning them jointly helps the network focus on the most informative areas of the fruit surface. Finally, sharing features helps the model generalize better, especially when the dataset is not very large.

#### 2.4.1. Multi-Task Loss Function

The model was trained end-to-end by optimizing a composite loss function that integrates the objectives of the five regression tasks (radius, color depth, color uniformity, injury area ratio, plumpness) and one classification task (color category). To dynamically balance the contribution of each task during training and avoid manual weight tuning, we employed a loss function based on homoscedastic uncertainty. The total loss is defined as follows:

$$L_{total}=\sum_{i=1}^{5}\left(\frac{1}{2\sigma_{i}^{2}}L_{i}^{reg}+log\sigma_{i}\right)\;+\;(\frac{1}{2\sigma_{cls}^{2}}L^{cls}+log\sigma_{cls})$$

Here, $L_{i}^{reg}$ denotes the mean squared error (MSE) loss for the i-th regression task, and $L_{i}^{reg}$ represents the cross-entropy loss for the color category classification. The parameters *σ_i_* and *σ_c__i__s_* are trainable variables that act as task-dependent weights, automatically adjusted by the model to reflect the relative uncertainty of each task.

#### 2.4.2. Training Methodology and Implementation

The multi-task network was trained using an end-to-end optimization strategy that simultaneously fine-tuned the shared AlexNet backbone and all six task-specific prediction heads. The training configuration employed the AdamW optimizer with an initial learning rate of 3 × 10^−4^ and weight decay of 1 × 10^−5^, parameters selected as follows: a preliminary grid search was conducted for the ResNet18 baseline over batch size (8, 16), number of epochs (50, 100, 150), and learning rate (3 × 10^−4^, 6 × 10^−4^), at a fixed input resolution of 256 × 256 and a 70:20:10 train/validation/test split, using validation-set performance as the selection criterion. The resulting configuration (learning rate = 3 × 10^−4^, batch size = 16, 100 epochs) was then held fixed across all eight backbones in the multi-backbone comparison (Section 3.3) to ensure a fair, controlled comparison; weight decay (1 × 10^−5^) was likewise fixed across all backbones. A ReduceLROnPlateau scheduler was implemented to dynamically adjust the learning rate when validation performance plateaued for three consecutive epochs, ensuring stable convergence across all quality prediction tasks.

The preprocessing pipeline was designed to maintain consistency and reproducibility across all experimental evaluations. All fruit images, cropped using YOLOv11-generated bounding boxes to isolate individual fruit regions, were resized to 256 × 256 pixels and normalized using ImageNet mean and standard deviation values. This standardized approach ensured that model comparisons were based on identical input conditions, eliminating potential biases from variable preprocessing techniques. The focus on consistent preprocessing rather than extensive augmentation allowed for more controlled comparisons between architectural variations. To enhance optimization stability in the multi-task learning framework, several technical strategies were incorporated. Gradient clipping with a maximum norm of 1.0 prevented gradient explosion during backpropagation, particularly important when balancing regression and classification objectives with different loss scales. The inherent dropout regularization within AlexNet’s fully connected layers (rate = 0.2) provided effective protection against overfitting. Task weights were not manually fixed but were learned automatically through the homoscedastic uncertainty-weighting scheme introduced in Section 2.4.1; during training, the learned weighting coefficient for the color classification task converged to approximately 2.0, reflecting its comparatively lower predictive uncertainty relative to the five regression tasks.

The training regimen extended for 100 epochs with a batch size of 16, utilizing a 70:20:10 dataset split for training, validation, and testing, respectively. This partitioning strategy ensured sufficient data for model optimization while maintaining robust evaluation metrics. Training progress was meticulously monitored through both task-specific loss components and comprehensive validation metrics, with model checkpoints saved based on optimal validation performance to facilitate reproducible results across experimental runs. To prevent data leakage, this split was performed at the source-image level: all fruit crops originating from the same original photograph (and therefore the same individual fruit) were assigned exclusively to one of the training, validation, or test subsets and never distributed across subsets; the same grouping principle was applied to the 1080/270 training/validation split used for YOLOv11 detection (Section 2.3.1).

#### 2.4.3. Evaluation Metrics

A comprehensive evaluation framework was established to assess model performance across all quality dimensions using task-specific metrics that aligned with practical grading requirements. For the five regression tasks predicting continuous quality attributes, three complementary metrics were employed: Mean Absolute Error (MAE) measured average prediction deviation, Root Mean Square Error (RMSE) emphasized larger errors, and the coefficient of determination (R^2^) quantified explained variance relative to expert annotations. Model performance was evaluated using task-appropriate indicators. MAE provided direct insight into prediction bias:

$$\mathrm{MAE}=\frac{1}{n}\sum_{j=1}^{n}|y_{j}-{\hat{y}}_{j}|$$

RMSE reflected prediction stability:

$$\mathrm{RMSE}=\sqrt{\frac{1}{n}\sum_{j=1}^{n}(y_{j}-{\hat{y}}_{j}{)}^{2}}$$

R^2^ measured explanatory power:

Briefly, MAE expresses the average absolute deviation between predicted and reference values in the original measurement unit of each trait, providing an intuitive, outlier-robust indicator of typical prediction error. RMSE also reports error in the original units but weights larger deviations more heavily owing to its squared-error formulation, making it more sensitive to occasional large mispredictions and therefore informative about worst-case reliability. R^2^ (coefficient of determination) is a scale-independent measure of the proportion of variance in the reference values explained by the model’s predictions, with values closer to 1 indicating better agreement; being dimensionless, R^2^ additionally enables comparison of predictive performance across traits expressed in different units (e.g., millimeters for radius versus dimensionless scores for color depth) [43,44].

$$R^{2}=1-\frac{\sum_{j=1}^{n}(y_{j}-{\hat{y}}_{j}{)}^{2}}{\sum_{j=1}^{n}(y_{j}-\bar{y}{)}^{2}}$$

For the color classification task, performance was assessed using:

$$\mathrm{Accuracy}=\frac{TP+TN}{TP+TN+FP+FN}$$

For the color classification task, performance was evaluated using classification accuracy, calculated as the percentage of correctly categorized fruit instances. This metric directly corresponded to practical grading applications where categorical color assessment is essential. All metrics were computed on the held-out test set following model convergence, ensuring unbiased performance estimation. The evaluation framework was consistently applied across all architectural comparisons, with the multi-task nature necessitating both individual task metrics and aggregated performance indicators.

## 3. Results

### 3.1. Detection Performance

**Figure 3:**
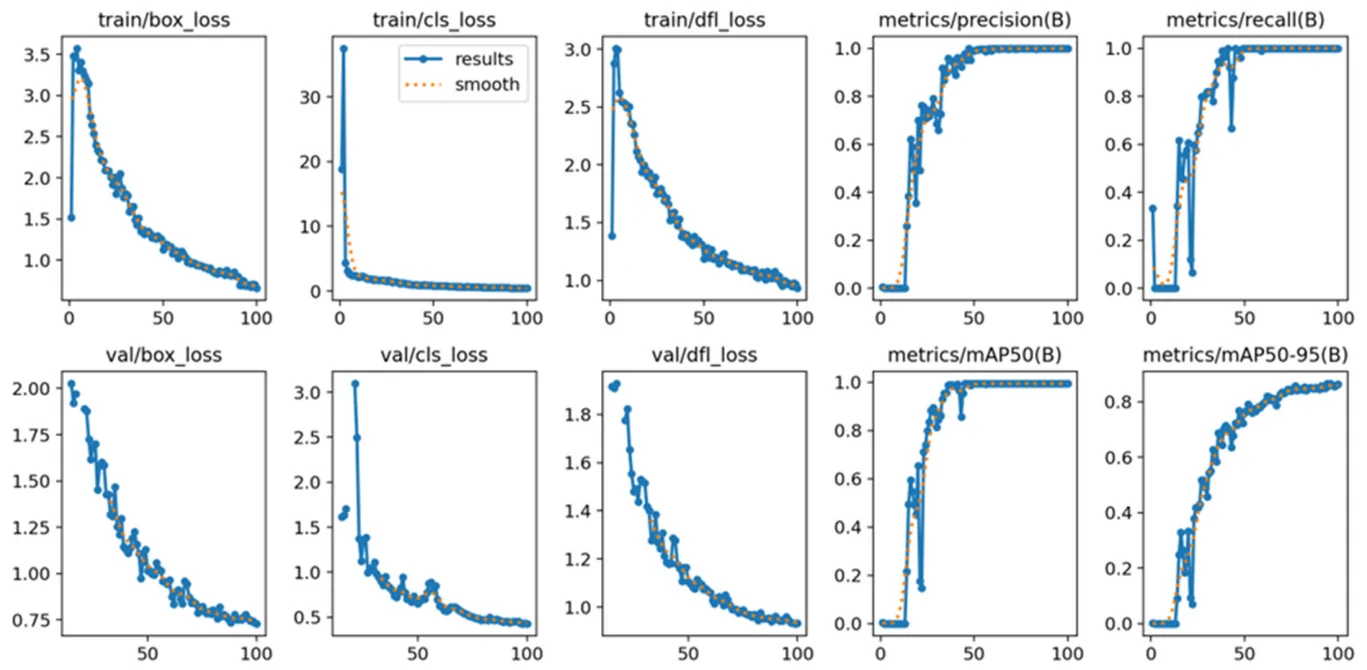
Training dynamics and detection performance of the YOLO-based waxberry detection model. Evolution of training and validation losses, including box loss, classification loss, and distribution focal loss (DFL), together with precision, recall, *mAP*@0.5, and *mAP*@0.5:0.95 over 100 epochs.

The performance of the YOLO-based Chinese bayberry detection model was first evaluated to ensure reliable extraction of individual waxberry instances for subsequent quality scoring. Figure 3 presents the training and validation dynamics over 100 epochs, including box loss, classification loss, distribution focal loss (DFL), and detection metrics.

As shown in Figure 3, all loss components exhibited a clear and stable downward trend during training. The box loss decreased steadily, indicating improved localization accuracy of the predicted bounding boxes. The classification loss dropped rapidly in the early epochs and converged to a low value, reflecting effective separation between Chinese bayberry instances and background regions. Similarly, the DFL showed consistent improvement, contributing to more precise boundary refinement. No obvious divergence between training and validation losses was observed, suggesting that the model did not suffer from severe overfitting despite the limited dataset size.

Detection accuracy metrics further confirm the robustness of the detection model. Precision and recall increased rapidly during the early stage of training and reached stable plateaus after approximately 40 epochs. The recall remained close to 1.00 throughout the later training stages, indicating that nearly all annotated waxberry instances were successfully detected. The mean Average Precision at an IoU threshold of 0.5 (*mAP*@0.5) reached 0.9950, while *mAP*@0.5:0.95 reached 0.8632 at the final epoch, reflecting strong localization performance under stricter overlap criteria. To further examine classification reliability, confusion matrices are shown in Figure S1a,b. The unnormalized confusion matrix indicates that 810 Chinese bayberry instances were correctly identified, with only three background regions misclassified as waxberry. The normalized confusion matrix shows near-perfect separation between waxberry and background classes. At the instance-count level, these results correspond to a precision of 0.9963 (810/813) and a recall of 1.00 for the waxberry class; the formal box-detection metrics logged during training (precision(B) = 0.9991, recall(B) = 1.0000) are consistent with this instance-level analysis, and both indicate high sensitivity and low false-negative rates.

Because the training records for this model specify task: detect (the standard Ultralytics YOLOv11 detection head, without an auxiliary mask branch) and the manual annotations consist of bounding boxes rather than polygon, brush, or pixel-level masks, mask-based segmentation metrics (mask *mAP*, mask IoU, Dice) are not available and are not reported. Table 1 summarizes the complete set of bounding-box detection metrics obtained from the training/validation logs. We have revised the manuscript throughout (Section 2.3, Section 2.4.2, Section 3.1 and Section 4.1) to consistently describe this stage as fruit detection rather than instance segmentation, and to clarify that downstream trait extraction uses bounding-box crops rather than segmentation masks. We also note that these metrics were computed on the 270-image validation split; a fully independent held-out detection test set (distinct from validation) was not established in this study, which we acknowledge as a limitation in Section 4.4.

Qualitative detection examples are presented in Figure S2a–f. The model accurately detected Chinese bayberry fruits of different sizes and color stages, while successfully ignoring background objects such as labels and containers. Bounding boxes were consistently aligned with berry boundaries, and detection confidence remained high across samples. These visual results confirm that the detected bounding-box crops are sufficiently accurate and stable to support downstream multi-task quality scoring. Overall, the YOLO-based detection model provides reliable instance-level extraction of Chinese bayberry fruits with high accuracy and strong generalization performance. This forms a solid foundation for subsequent quality evaluation using the multi-task scoring network.

### 3.2. Quality Scoring Performance

#### 3.2.1. Training Convergence and Task-Wise Loss Behavior

The training behavior of the AlexNet-based multi-task quality scoring model is summarized in Figure S3. Across all tasks, training losses decreased rapidly during the initial stage and gradually stabilized after approximately 40 epochs. Validation losses followed similar trajectories and remained close to the corresponding training curves throughout the optimization process, indicating stable convergence under the multi-task learning setting. For color-related attributes, the model showed fast and consistent convergence. Both color depth and color uniformity losses declined sharply during early training and reached low, stable levels, with only minor fluctuations in later epochs. The color classification task exhibited a steady reduction in loss and maintained a small gap between training and validation curves, suggesting that discriminative color features were learned with good generalization.

The regression tasks associated with global morphological characteristics demonstrated clear convergence patterns. Radius loss decreased rapidly and stabilized at a low value, reflecting an accurate estimation of fruit size from appearance cues. Plumpness loss followed a similar trend, with training and validation losses becoming closely aligned after convergence. This behavior suggests that the model effectively captured global shape-related features that are consistently expressed across samples. In contrast, the surface damage regression task showed higher loss values and greater variability during training. Compared with size- and color-related attributes, surface injuries are often localized and visually heterogeneous, which increases prediction difficulty. Despite these challenges, the validation loss remained stable and did not diverge from the training loss, indicating that the model retained reasonable generalization ability for this more complex quality attribute.

#### 3.2.2. Regression Accuracy and Fitting Performance of Quality Traits

**Figure 4:**
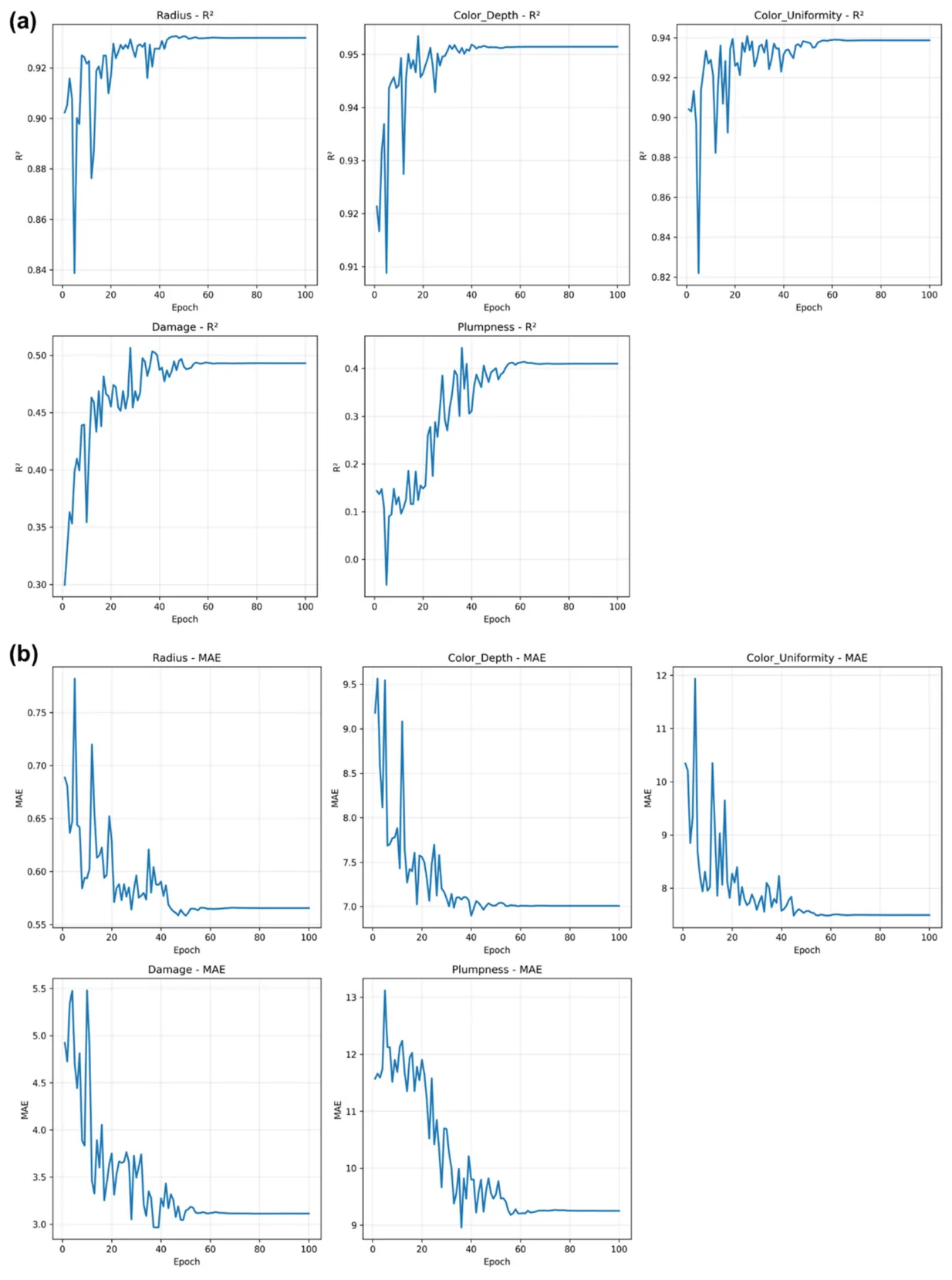

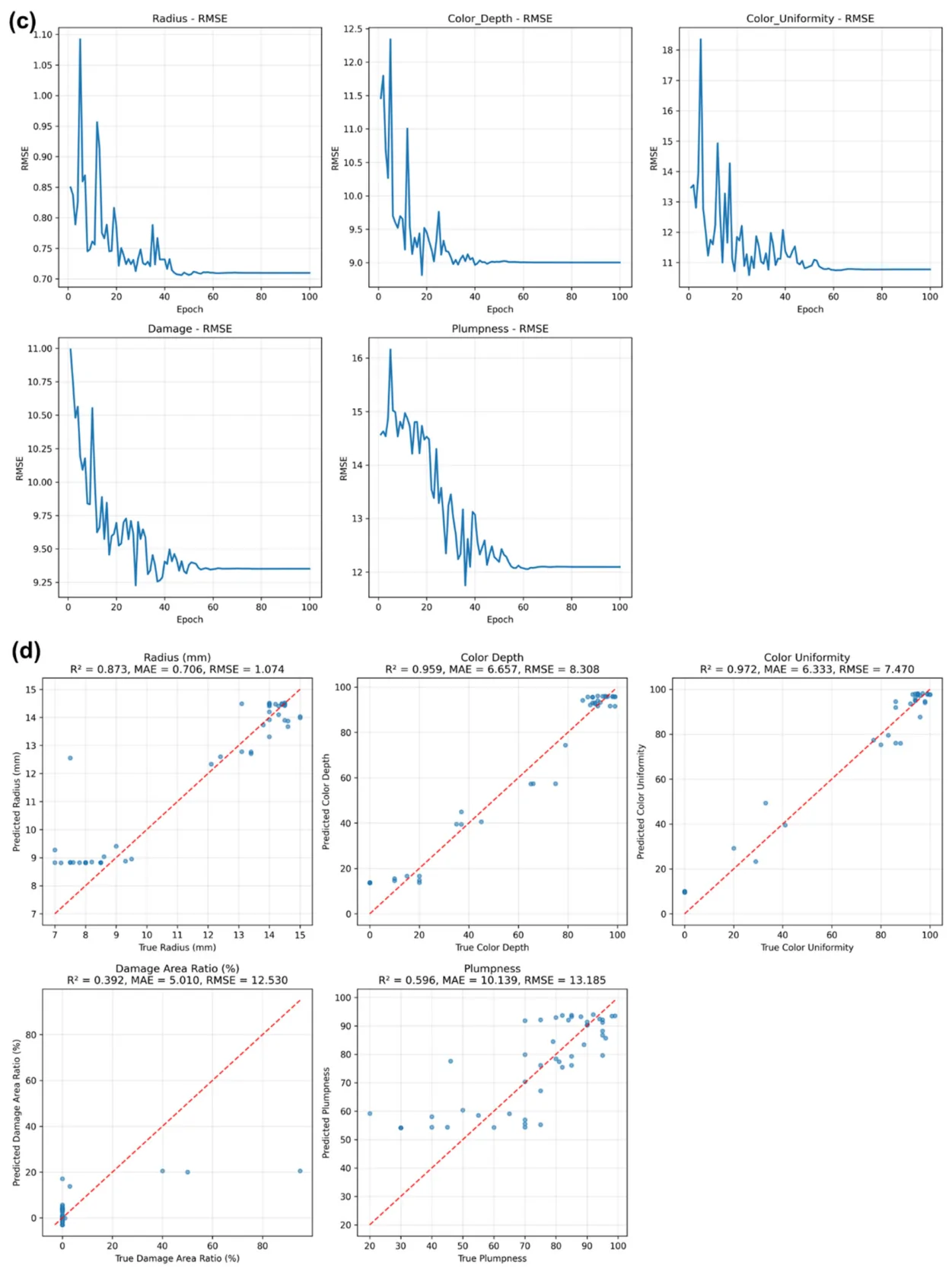
Regression performance of the AlexNet-based multi-task quality scoring model. Training evolution of R^2^ (**a**), MAE (**b**), and RMSE (**c**) for radius, color depth, color uniformity, surface damage ratio, and plumpness, together with scatter plots comparing predicted and reference values for each quality trait (**d**).

The regression performance of the AlexNet-based model for the five continuous quality traits, including radius, color depth, color uniformity, surface damage ratio, and plumpness, is shown in Figure 4. The evolution of the coefficient of determination (R^2^), mean absolute error (MAE), and root mean square error (RMSE) over training epochs provides a detailed view of task-wise learning behavior. For radius prediction, the model achieved rapid improvement in fitting accuracy. As shown in Figure 4a, the R^2^ value increased steadily and stabilized at a high level, while both MAE and RMSE decreased consistently during training. The scatter plot in Figure 4d shows a close alignment between predicted and reference radius values, indicating reliable estimation of fruit size.

Similar convergence patterns were observed for color-related traits. The regression results for color depth and color uniformity exhibited high R^2^ values and low error metrics after convergence, reflecting accurate modeling of pigment intensity and spatial color consistency across the fruit surface. The corresponding scatter plots demonstrate strong linear relationships between predicted and true values, with most samples distributed near the 1:1 reference line. In contrast, the surface damage ratio presented greater variability. Although the R^2^ value increased during training, it remained lower than that of other traits, and both MAE and RMSE showed larger residual values. This outcome is expected because surface damage is often localized, irregular, and more sensitive to annotation uncertainty than global traits such as size or color. Nevertheless, the error curves stabilized after convergence, suggesting that the model learned consistent patterns despite the higher task difficulty.

Plumpness regression showed intermediate performance between geometric and damage-related traits. The R^2^ curve increased gradually and stabilized at a moderate level, while error metrics declined steadily. The scatter plot indicates a reasonable correspondence between predicted and reference plumpness values, with some dispersion remaining for samples at the extremes of the scale.

#### 3.2.3. Test-Set Numerical Performance Summary

Table 2 reports exact test-set performance (*n* = 48 held-out test samples) for each of the six quality traits, computed from the saved AlexNet test-result files: MAE, RMSE, and R^2^ for the five regression traits, and accuracy/F1-score for color classification. Only a single training/evaluation run was preserved, so repeated-run variability and confidence intervals are not available and are not reported; for single-label multi-class classification, micro-F1 is equivalent to accuracy (0.9792, 47/48 correctly classified).

#### 3.2.4. Comparison with Classical Geometric Baselines

Because plumpness is, in principle, related to fruit shape and could be approximated directly from image geometry, we examined a naive geometric baseline for comparison. A mask-based geometric baseline (using pixel-level segmentation masks) could not be constructed, since no pixel-level masks were annotated in this study (Section 2.3). As an internal check, we instead computed a rough baseline directly from the bounding-box crop aspect ratio across all 476 matched cropped fruit images for which a crop and reference plumpness score could be paired; because this set is not identical to, and not strictly matched with, the 48-sample held-out test set used for the deep model, it should be read as an approximate internal check rather than a rigorous test-set baseline. This naive crop-aspect-ratio baseline achieved MAE = 18.1128, RMSE = 24.9164, and R^2^ = −0.4197 for plumpness, substantially worse than the multi-task deep model’s test-set performance for the same trait (MAE = 10.1393, RMSE = 13.1845, R^2^ = 0.5956; Table 2), including a negative R^2^ indicating that the naive baseline performs worse than simply predicting the mean. This comparison, while approximate, supports the added value of the learned multi-task approach over a direct, uncalibrated geometric calculation from the raw crop.

### 3.3. Comparison of Multi-Task Quality Scoring Performance Across Backbone Models

**Figure 5:**
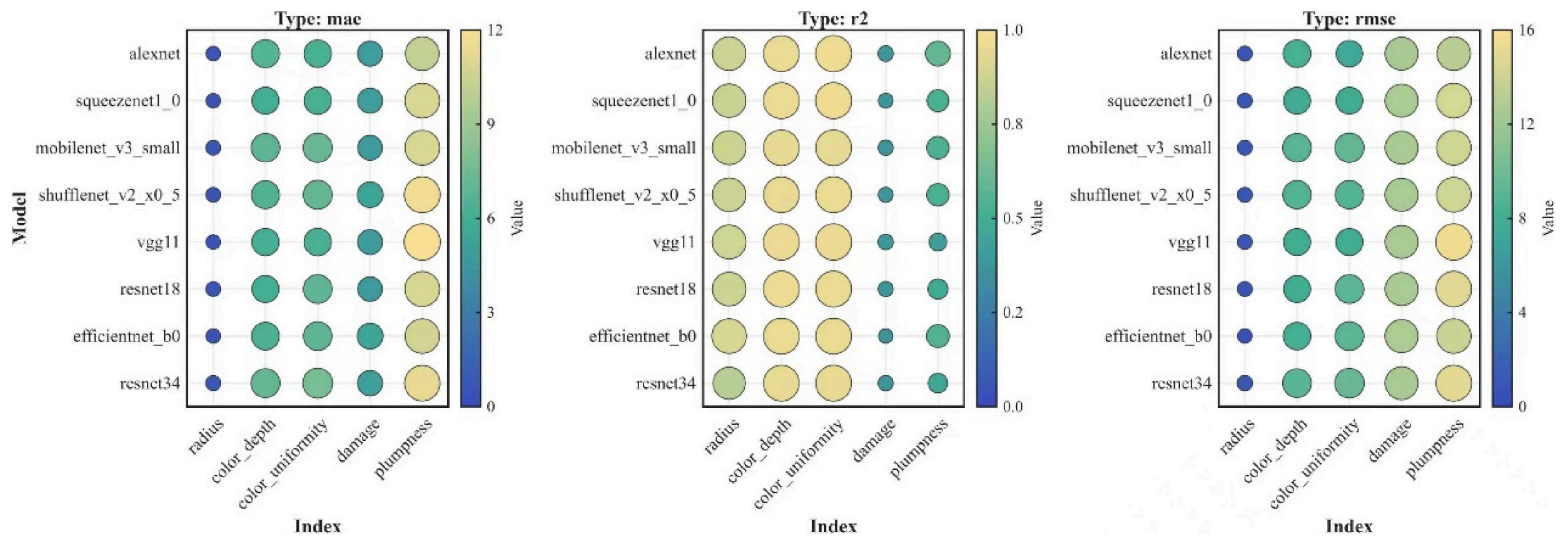
Comparison of multi-task regression performance across different backbone models. Bubble plots of MAE, R^2^, and RMSE for radius, color depth, color uniformity, surface damage ratio, and plumpness, evaluated using AlexNet, SqueezeNet, MobileNetV3-Small, ShuffleNetV2, VGG11, ResNet18, ResNet34, and EfficientNet-B0.

The performance of the AlexNet-based multi-task model was further compared with several commonly used convolutional backbones, including SqueezeNet, MobileNetV3-Small, ShuffleNetV2, VGG11, ResNet18, ResNet34, and EfficientNet-B0. Figure 5 summarizes the comparison results using three evaluation metrics—MAE, R^2^, and RMSE—across five regression-based quality traits.

For size-related and color-related attributes, most models achieved relatively high prediction accuracy. Radius, color depth, and color uniformity exhibited consistently low MAE values and high R^2^ scores across different backbones. Among them, AlexNet showed competitive or superior performance, with low error levels and stable fitting quality, particularly for radius estimation and color uniformity. Lightweight architectures, such as MobileNetV3 and ShuffleNetV2, achieved comparable results for these simpler traits, but their performance showed slightly higher variability.

Clearer differences emerged for more complex quality attributes. For surface damage ratio, all models exhibited reduced R^2^ values and increased error metrics, reflecting the difficulty of modeling localized and irregular surface defects. Despite this challenge, AlexNet maintained relatively lower MAE and RMSE values compared with several deeper or more compact architectures, indicating better robustness for this task. A similar pattern was observed for plumpness prediction, where AlexNet achieved higher R^2^ and lower error values than deeper residual networks such as ResNet34 and EfficientNet-B0.

Deeper architectures did not consistently outperform shallower models in this multi-task setting. While ResNet and EfficientNet variants showed strong performance for certain traits, their overall error levels were not uniformly lower than those of AlexNet. This suggests that increased depth or parameter count does not necessarily translate into better performance for small-scale, multi-attribute fruit quality datasets. The comparison results indicate that AlexNet provides a favorable balance between model capacity and generalization ability across multiple quality traits. Its stable performance across both global attributes (size and color) and more heterogeneous traits (surface damage and plumpness) supports its suitability as the backbone network for multi-task Chinese bayberry quality scoring.

In terms of model complexity, parameter counts were computed directly from the implemented multi-task architectures (backbone plus task-specific heads), and approximate computational cost was estimated as GMACs for a 256 × 256 input (Table 3). AlexNet, at 60,202,891 parameters, is the largest of the eight backbones by parameter count, considerably larger than the lightweight architectures (SqueezeNet, MobileNetV3-Small, ShuffleNetV2-x0.5: 1.2–2.0 M parameters) and even larger than VGG11 (10.2 M) and the ResNet variants (12.2–22.3 M). Despite this, AlexNet’s estimated computational cost (0.93 GMACs) is comparable to or lower than several smaller-parameter models, such as SqueezeNet (1.07 GMACs) and VGG11 (10.00 GMACs), reflecting its simple, non-residual convolutional structure. We do not report measured inference latency, since no controlled inference-time logs under a fixed hardware/software environment were preserved for this study; to avoid overclaiming, we therefore distinguish computed parameter counts and estimated FLOPs from (unmeasured) latency, and recommend that deployment-oriented latency benchmarking be performed directly on the target hardware in future work.

## 4. Discussion

### 4.1. Robust Instance Extraction for Chinese Bayberry Quality Assessment

The first stage of the proposed framework focuses on reliable detection of individual Chinese bayberry instances. High recall and stable *mAP* values indicate that the YOLO-based model successfully captured nearly all annotated fruits, providing clean instance crops for downstream quality scoring. This step is critical, as errors at the detection stage would directly propagate into the multi-task regression and classification results. Compared with earlier fruit detection studies, the detection accuracy achieved here is consistent with or exceeds reported performance for apples, citrus, and tomatoes under controlled or semi-controlled imaging conditions [41,45]. Chinese bayberry poses additional challenges due to its dark color range, fine surface texture, and limited contrast with the background. The results suggest that modern single-stage detectors are sufficiently robust to handle these characteristics and can support more advanced tasks beyond fruit counting, such as quality evaluation and grading.

A key revision in this study is the replacement of the ResNet18-based scoring backbone with AlexNet. Although ResNet architectures are widely used for image recognition tasks, their advantages are most evident in large-scale datasets where deep hierarchical representations are required [42]. In contrast, the quality scoring problem addressed here relies primarily on global appearance cues, such as size, color distribution, and coarse surface texture. AlexNet, with its shallower structure and lower parameter complexity, appears better matched to the scale and variability of the available dataset [46,47,48]. The improved performance suggests that deeper networks may introduce unnecessary capacity, increasing the risk of overfitting when training data are limited, or labels contain subjective noise [49]. Similar observations have been reported in agricultural vision studies, where simpler or moderately deep networks achieved better generalization than deeper alternatives under constrained data conditions [36,37].

Mechanistically, the quality-scoring task in this study depends primarily on coarse, globally distributed appearance cues (overall size, color distribution, and coarse surface texture) rather than the fine-grained hierarchical representations that deeper residual or efficient architectures are designed to exploit. With only 1350 source images and labels partly derived from subjective expert scoring, deeper networks with substantially larger parameter counts are more prone to overfitting and to fitting label noise rather than generalizable signal. AlexNet’s shallower, non-residual design and its relatively strong built-in dropout regularization in the fully connected layers appear better matched to this combination of limited sample size, moderate label noise, and globally expressed target attributes, consistent with reports that moderately deep networks can outperform deeper alternatives under constrained agricultural data conditions [35,36].

### 4.2. Interpretation of Performance Differences Across Quality Traits

The multi-task learning results reveal clear differences in predictability among quality attributes. Size-related and color-related traits, including radius, color depth, and color uniformity, achieved higher R^2^ values and lower error metrics. These attributes are strongly expressed in RGB appearance and are therefore more accessible to convolutional feature extraction.

In contrast, surface injury area ratio and plumpness were more difficult to predict across all tested models. Surface damage is often localized and irregular, and mild injuries may visually resemble natural color variation. Plumpness reflects three-dimensional shape characteristics that cannot be fully captured from a single two-dimensional view. Similar limitations have been reported in fruit grading and defect detection studies, where defect severity and shape-related attributes consistently showed lower predictive accuracy than size or color metrics [38,39]. The use of uncertainty-weighted multi-task loss helps balance these heterogeneous tasks by adapting loss contributions during training. This strategy reduces reliance on manual tuning and has been shown to improve stability when combining regression and classification objectives with different scales and noise levels [50].

These limitations are consistent with the intrinsic constraints of RGB-based feature extraction. A single-view RGB image encodes only two-dimensional color and texture information; it cannot directly resolve the three-dimensional volumetric cues that fully determine plumpness, which is here approximated by a two-dimensional aspect ratio proxy rather than true volumetric fullness. Similarly, surface injury is a localized, low-contrast, and highly variable signal that can closely resemble natural pigment variation in color space, making it inherently harder for globally pooled convolutional features to localize precisely than holistic attributes such as overall color or size. Complementary modalities (e.g., depth or hyperspectral imaging) or higher-resolution local feature extraction may be required to further improve prediction of these two traits.

### 4.3. Model Comparison and Practical Considerations for Deployment

The comparison across multiple backbone architectures highlights that higher model complexity does not guarantee better performance for multi-task fruit quality scoring. While deeper or more recent networks, such as ResNet34 and EfficientNet-B0, performed well for certain traits, they did not consistently outperform AlexNet across all metrics. Lightweight models, such as MobileNet and ShuffleNet, showed competitive results for some attributes but exhibited greater variability. These findings emphasize the importance of aligning model capacity with dataset size and application requirements. In practical deployment scenarios, such as packing lines or on-device grading systems, inference speed, memory footprint, and training stability are as important as accuracy. The consistent performance of AlexNet across multiple traits suggests that it offers a favorable balance between accuracy and efficiency for real-world Chinese bayberry quality assessment.

### 4.4. Limitations and Future Implications

Several limitations should be addressed in future work. First, the dataset size and acquisition conditions remain constrained, which may limit generalization across orchards, seasons, or imaging devices. Expanding the dataset to include field-acquired images under varying illumination and background conditions would improve robustness. Additionally, we will explore domain adaptation techniques, such as unsupervised domain adaptation and test-time adaptation, to bridge the domain gap between training and deployment environments with minimal annotation cost. Second, although the current labels were annotated by three experts with inter-rater agreement assessed to ensure consistency, expert judgment inevitably introduces certain subjectivity. To further mitigate this limitation and enhance the reliability of the dataset, we plan to involve additional trained experts in future annotation rounds. This multi-rater expansion will not only strengthen the label quality, but also allow for a more comprehensive quantification of annotation uncertainty. From an application perspective, the proposed detection–scoring pipeline provides a flexible foundation for automated grading systems. It could be integrated into conveyor-based sorting lines for real-time quality evaluation or adapted for portable imaging devices to support on-site assessment by growers and traders. Future extensions may also explore multimodal data, such as depth or spectral information, to improve the prediction of shape-related and internal quality attributes. The modular design of the framework facilitates such extensions without requiring complete system retraining. Because all training and evaluation images were acquired under fixed, uniform laboratory illumination and a single camera viewpoint, the performance reported here represents an upper bound under controlled conditions; variable natural or artificial illumination, shadowing, fruit orientation, and cluttered backgrounds in real orchard or packing-line environments are expected to reduce both detection and scoring accuracy, and field validation or illumination-normalization preprocessing would be needed prior to practical deployment.

## Figures and Tables

**Table 1:** Bounding-box detection metrics for the YOLOv11 waxberry detector (270-image validation split).

Metric	Value
Precision (B)	0.9991
Recall (B)	1.0000
*mAP*50 (B)	0.9950
*mAP*50-95 (B)	0.8632

**Table 2:** Exact test-set performance (*n* = 48 held-out test samples) for each of the six quality traits.

Trait	MAE	RMSE	R^2^/Accuracy	F1-Score (Color Only)	Notes
Radius (mm)	0.7064	1.0735	0.8735		Test set *n* = 48
Color depth	6.6568	8.3083	0.9589		
Color uniformity	6.3329	7.4702	0.9717		
Surface injury area ratio	5.0100	12.5305	0.3918		
Plumpness	10.1393	13.1845	0.5956		
Color classification (categorical)	—	—	Accuracy = 0.9792 (47/48)	micro-F1 = 0.9792	Single run; repeated-run variability not computed

**Table 3:** Parameter counts and approximate computational cost (GMACs at 256 × 256 input) for the eight compared backbones.

Backbone	Parameters	Approx. GMACs @ 256 × 256
AlexNet	60,202,891	0.93
SqueezeNet1.0	1,181,963	1.07
MobileNetV3-Small	2,016,683	0.08
ShuffleNetV2-x0.5	1,181,547	0.06
VGG11	10,211,851	10.00
ResNet18	12,167,883	2.38
EfficientNet-B0	6,178,567	0.51
ResNet34	22,276,043	4.81

## Data Availability

Data will be made available on request. The source code used in this study has been permanently archived and is publicly available at Zenodo: https://doi.org/10.5281/zenodo.21760021.

## Supplementary Materials

The following supporting information can be downloaded at: https://www.mdpi.com/article/10.3390/foods15162852/s1, Figure S1: Confusion matrix analysis of waxberry detection results. (a) Confusion matrix showing prediction counts for waxberry and background classes. (b) Normalized confusion matrix illustrating class-wise prediction accuracy. Figure S2: Qualitative results of waxberry detection and localization. Representative detection examples under different color stages and illumination conditions. Figure S3: Multi-task training convergence of the AlexNet-based quality scoring model. Loss convergence for six quality attributes, including color depth, color category, color uniformity, surface damage ratio, plumpness, and radius, over 100 training epochs.

## References

[1] Fan J.C.F., He H.L., Liu S.Y., Ren R., Wang S.T. (2022). Investigation of Pesticide Residues in *Fragaria* and *Myrica rubra* Sold in Hangzhou. J. Food Prot..

[2] Zhang Q., Huang Z., Wang Y., Wang Y., Fu L., Su L. (2021). Chinese bayberry (*Myrica rubra*) phenolics mitigated protein glycoxidation and formation of advanced glycation end-products: A mechanistic investigation. Food Chem..

[3] Zhu Y., Jiang J., Yue Y., Feng Z., Chen J., Ye X. (2020). Influence of mixed probiotics on the bioactive composition, antioxidant activity and appearance of fermented red bayberry pomace. LWT.

[4] Zhang Y., Pan H., Ye X., Chen S. (2022). Proanthocyanidins from Chinese bayberry leaves reduce obesity and associated metabolic disorders in high-fat diet-induced obese mice through a combination of AMPK activation and an alteration in gut microbiota. Food Funct..

[5] Saeed M., Elsadek M.A., Chen Z., Zhao L., Wang G., Zhou C., Sun D., Gao Z., Jiao Y. (2025). Enhancing the terpenoid and flavonoid profiles and fruit quality in an elite Chinese bayberry line through hybridization. Food Chem..

[6] Zhu Y., Wang M., Zhu J., Zhang X., Ye X., Chen J. (2024). Protective effects of Chinese bayberry pomace wine against oxidative stress on Drosophila melanogaster. Food Res. Int..

[7] Fu Y., Zheng Y., Shi B., Liu B., Zhang M., Zhu Y. (2025). Green extraction of anthocyanins from Chinese bayberry (*Myrica rubra* Sieb. Et Zucc.) using acidic deep eutectic solvents: Enhancing the physicochemical properties and stability of anthocyanins. Food Res. Int..

[8] Suo K., Zhang Y., Feng Y., Yang Z., Zhou C., Chen W., Wang J. (2023). Ultrasonic synergistic slightly acidic electrolyzed water processing to improve postharvest storage quality of Chinese bayberry. Ultrason Sonochem..

[9] Olimi E., Kusstatscher P., Wicaksono W.A., Abdelfattah A., Cernava T., Berg G. (2022). Insights into the microbiome assembly during different growth stages and storage of strawberry plants. Environ. Microbiome.

[10] Shi L., Chen X., Wang K., Yang M., Chen W., Yang Z., Cao S. (2021). MrMYB6 From Chinese Bayberry (*Myrica rubra*) Negatively Regulates Anthocyanin and Proanthocyanidin Accumulation. Front Plant Sci..

[11] Zhou Z., Zahid U., Majeed Y., Nisha, Mustafa S., Sajjad M.M., Butt H.D., Fu L. (2023). Advancement in artificial intelligence for on-farm fruit sorting and transportation. Front Plant Sci..

[12] Bauer A., Bostrom A.G., Ball J., Applegate C., Cheng T., Laycock S., Rojas S.M., Kirwan J., Zhou J. (2019). Combining computer vision and deep learning to enable ultra-scale aerial phenotyping and precision agriculture: A case study of lettuce production. Hortic. Res..

[13] Nithya R., Santhi B., Manikandan R., Rahimi M., Gandomi A.H. (2022). Computer Vision System for Mango Fruit Defect Detection Using Deep Convolutional Neural Network. Foods.

[14] Neupane C., Walsh K.B., Goulart R., Koirala A. (2024). Developing Machine Vision in Tree-Fruit Applications-Fruit Count, Fruit Size and Branch Avoidance in Automated Harvesting. Sensors.

[15] Nguyen N.H., Michaud J., Mogollon R., Zhang H., Hargarten H., Leisso R., Torres C.A., Honaas L., Ficklin S. (2024). Rating pome fruit quality traits using deep learning and image processing. Plant Direct.

[16] Ni X., Li C., Jiang H., Takeda F. (2020). Deep learning image segmentation and extraction of blueberry fruit traits associated with harvestability and yield. Hortic. Res..

[17] Wang C., Han Q., Li C., Zou T., Zou X. (2024). Fusion of fruit image processing and deep learning: A study on identification of citrus ripeness based on R-LBP algorithm and YOLO-CIT model. Front Plant Sci..

[18] Zhang Y., Struckmeyer S., Kolb A., Reichardt S. (2026). Tomato Multi-Angle Multi-Pose Dataset for Fine-Grained Phenotyping. Sci. Data.

[19] Liu Y., Yu Q., Geng S., Guo S., Liu L. (2025). SSViT-YOLOv11: Fusing lightweight YOLO & ViT for coffee fruit maturity detection. Front Plant Sci..

[20] Ji J., Lu X., Ma H., Lu X., Yang Y., Cui H., Yu M., Xie X. (2026). Accurate detection and density estimation of peach tree inflorescences using an improved YOLOv11 model. Front Plant Sci..

[21] Wu X., Su X., Ma Z., Xu B. (2025). YOLO-lychee-advanced: An optimized detection model for lychee pest damage based on YOLOv11. Front Plant Sci..

[22] Hadipour-Rokni R., Askari Asli-Ardeh E., Jahanbakhshi A., Esmaili Paeen-Afrakoti I., Sabzi S. (2023). Intelligent detection of citrus fruit pests using machine vision system and convolutional neural network through transfer learning technique. Comput Biol. Med..

[23] Li C., Jin C., Zhai Y., Pu Y., Qi H., Zhang C. (2025). Simultaneous detection of citrus internal quality attributes using near-infrared spectroscopy and hyperspectral imaging with multi-task deep learning and instrumental transfer learning. Food Chem..

[24] Krishnan N.M., Kumar S., Panda B. (2024). Fruit-In-Sight: A deep learning-based framework for secondary metabolite class prediction using fruit and leaf images. PLoS ONE.

[25] Agarla M., Napoletano P., Schettini R. (2023). Quasi Real-Time Apple Defect Segmentation Using Deep Learning. Sensors.

[26] Yu F., Lu T., Xue C. (2023). Deep Learning-Based Intelligent Apple Variety Classification System and Model Interpretability Analysis. Foods.

[27] Sun Z., Tian H., Hu D., Yang J., Xie L., Xu H., Ying Y. (2025). Integrating deep learning and data fusion for enhanced oranges soluble solids content prediction using machine vision and Vis/NIR spectroscopy. Food Chem..

[28] Lee D., Oh J., Choi Y., Lee D., Lee H., Shim S.B., Ha Y. (2023). Development of YOLO-based apple quality sorter. Korean J. Agric. Sci..

[29] Zheng H., Sun L., Wang Y., Yang H., Zhang S. (2025). Image-Based Detection of Chinese Bayberry (*Myrica rubra*) Maturity Using Cascaded Instance Segmentation and Multi-Feature Regression. Horticulturae.

[30] Bochkovskiy A., Wang C.Y., Liao H.Y.M. (2020). YOLOv4: Optimal Speed and Accuracy of Object Detection. arXiv.

[31] Loshchilov I., Hutter F. Decoupled weight decay regularization. Proceedings of the International Conference on Learning Representations (ICLR).

[32] Li C., Li L., Jiang H., Weng K., Geng Y., Li L., Qiao Y. (2020). Generalized focal loss: Learning qualified and distributed bounding boxes for dense object detection. Adv. Neural Inf. Process. Syst..

[33] Everingham M., Van Gool L., Williams C.K.I., Winn J., Zisserman A. (2010). The Pascal Visual Object Classes (VOC) Challenge. Int. J. Comput Vis..

[34] Zheng Z., Wang P., Liu W., Li J., Ye R., Ren D. (2020). Distance-IoU loss: Faster and better learning for bounding box regression. Proc. AAAI Conf. Artif. Intell..

[35] Dawood P., Breuer F., Gram M., Homolya I., Jakob P.M., Zaiss M., Blaimer M. (2025). Image space formalism of convolutional neural networks for k-space interpolation. Magn. Reson Med..

[36] Shorten C., Khoshgoftaar T.M. (2019). A survey on image data augmentation for deep learning. J. Big Data.

[37] Bonora A., Bortolotti G., Bresilla K., Corelli Grappadelli L., Manfrini L. (2021). A convolutional neural network approach to detecting fruit physiological disorders and maturity in ‘Abbé Fétel’ pears. Biosyst. Eng..

[38] Bantan R.A.R., Ali A., Naeem S., Jamal F., Elgarhy M., Chesneau C. (2020). Discrimination of sunflower seeds using multispectral and texture dataset in combination with region selection and supervised classification methods. Chaos.

[39] Wan P., Toudeshki A., Tan H., Ehsani R. (2020). A methodology for fresh fruit quality evaluation using computer vision. Comput. Electron. Agric..

[40] Habib A., Karmakar C., Yearwood J. (2023). Interpretability and Optimisation of Convolutional Neural Networks Based on Sinc-Convolution. IEEE J. Biomed. Health Inform..

[41] Fu L., Feng Y., Wu J., Liu Z., Majeed Y. (2022). Fruit detection and quality assessment based on deep learning: A review. Comput. Electron. Agric..

[42] He K., Zhang X., Ren S., Sun J. (2016). Deep residual learning for image recognition. Proceedings of the IEEE Conference on Computer Vision and Pattern Recognition.

[43] Ashtiani S., Manafiazar G., Do D., Hu G., Davoudi P., Miar Y. (2026). Fuzzy-augmented machine learning models for the prediction of feed efficiency and disease status in livestock: A mink case study. Smart Agric. Technol..

[44] Chai T., Draxler R.R. (2014). Root mean square error (RMSE) or mean absolute error (MAE)?—Arguments against avoiding RMSE in the literature. Geosci. Model Dev..

[45] Wang C., Liu S., Wang Y., Xiong J., Zhang Z., Zhao B., Luo L., Lin G., He P. (2022). Application of Convolutional Neural Network-Based Detection Methods in Fresh Fruit Production: A Comprehensive Review. Front Plant Sci..

[46] Safarov F., Kutlimuratov A., Khojamuratova U., Abdusalomov A., Cho Y.I. (2025). Enhanced AlexNet with Gabor and Local Binary Pattern Features for Improved Facial Emotion Recognition. Sensors.

[47] Mishra U., Pandey A., G L., K T. (2025). Deep learning-based disease detection in potato and mango leaves: A comparative study of CNN, AlexNet, ResNet, and EfficientNet. Sci. Rep..

[48] Miranda V.L., Oliveira-Correia J.P.S., Galvão C., Obara M.T., Peterson A.T., Gurgel-Gonçalves R. (2025). Automated identification of Chagas disease vectors using AlexNet pre-trained convolutional neural networks. Med. Vet. Entomol..

[49] Martínez Pérez J.A., Pérez Martín P.S. (2024). Regresión logística [Logistic regression]. Semergen.

[50] Kendall A., Gal Y., Cipolla R. (2018). Multi-task learning using uncertainty to weigh losses for scene geometry and semantics. Proceedings of the IEEE Conference on Computer Vision and Pattern Recognition.

