# Supplementary material for: Image-Based Assessment of External Quality Traits in Chinese Bayberry (Myrica rubra) Using a Deep Learning Detection and Multi-Trait Scoring Framework
Source: Foods. 2026 Aug 15;15(16):2852. doi: 10.3390/foods15162852 (PMC13512740; doi:10.3390/foods15162852)
Supplement: Supplementary file 1 [file foods-15-02852-s001.zip › foods-4370274-supplementary.pdf]

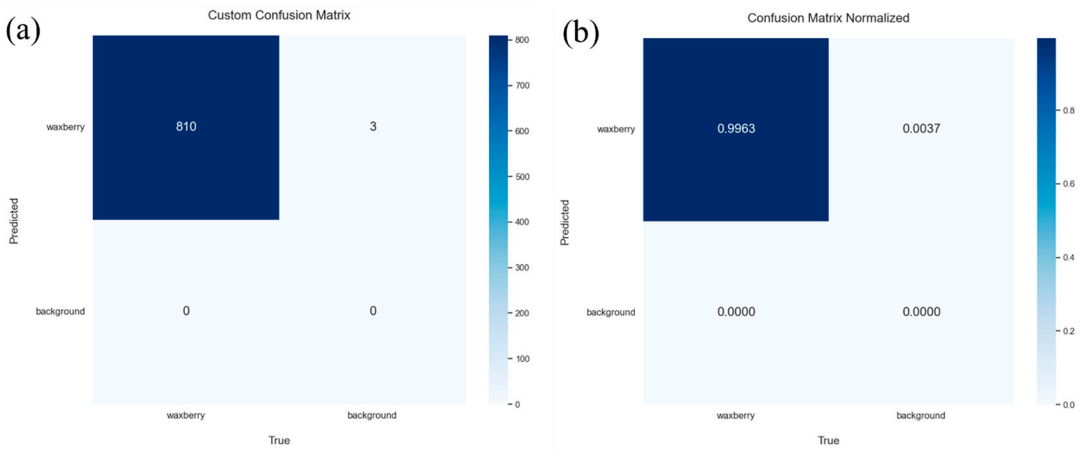

Figure S1. Confusion matrix analysis of waxberry detection results.

(a) Confusion matrix showing prediction counts for waxberry and background classes.

(b) Normalized confusion matrix illustrating class-wise prediction accuracy.

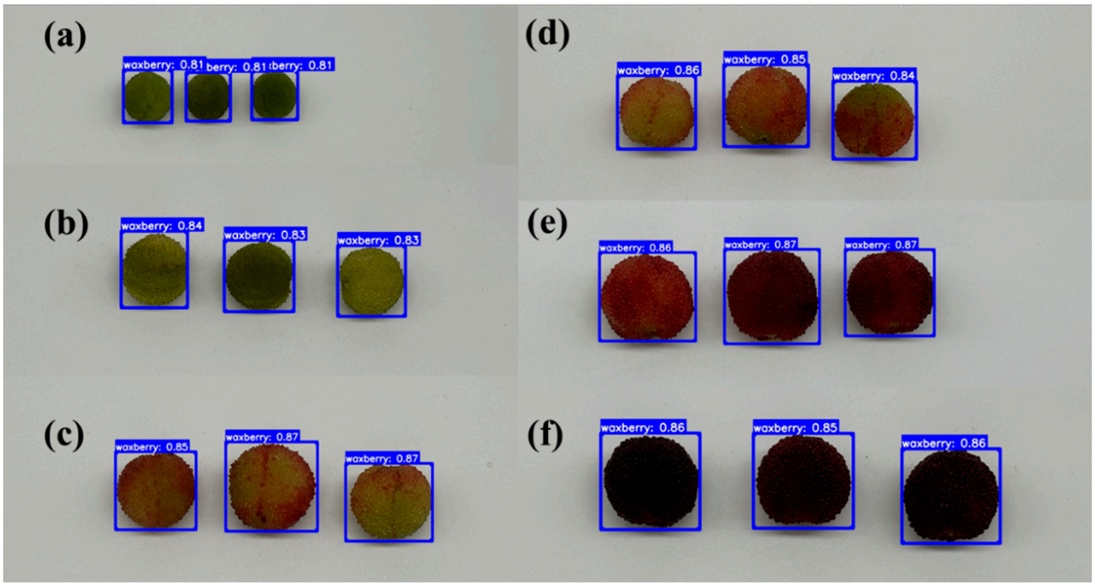

Figure S2. Qualitative results of waxberry detection and localization.

Representative detection examples under different color stages and illumination conditions.

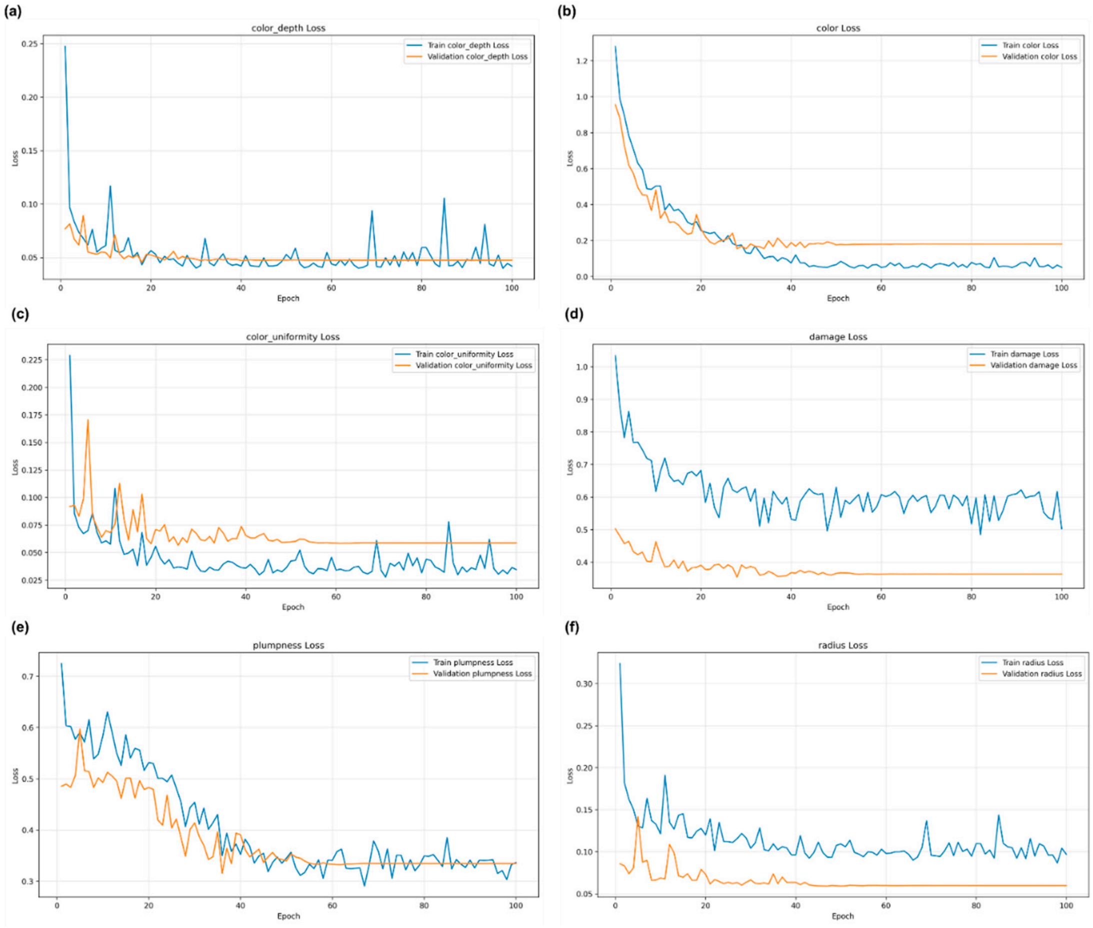

Figure S3. Multi-task training convergence of the AlexNet-based quality scoring model.

Loss convergence for six quality attributes, including color depth, color category, color uniformity, surface damage ratio, plumpness, and radius, over 100 training epochs.
